# lncRNA-Six1 Is a Target of miR-1611 That Functions as a ceRNA to Regulate Six1 Protein Expression and Fiber Type Switching in Chicken Myogenesis

**DOI:** 10.3390/cells7120243

**Published:** 2018-12-04

**Authors:** Manting Ma, Bolin Cai, Liang Jiang, Bahareldin Ali Abdalla, Zhenhui Li, Qinghua Nie, Xiquan Zhang

**Affiliations:** 1 Department of Animal Genetics, Breeding and Reproduction, College of Animal Science, South China Agricultural University, Guangzhou 510642, Guangdong, China; mamanting@stu.scau.edu.cn (M.M.); bolincai@stu.scau.edu.cn (B.C.); jiangliangscau@163.com (L.J.); abdalla406@163.com (B.A.A.); lizhenhui@scau.edu.cn (Z.L.); xqzhang@scau.edu.cn (X.Z.); 2 Guangdong Provincial Key Lab of Agro-Animal Genomics and Molecular Breeding, and Key Laboratory of Chicken Genetics, Breeding and Reproduction, Ministry of Agriculture, Guangzhou 510642, Guangdong, China; 3 National-Local Joint Engineering Research Center for Livestock Breeding, Guangzhou 510642, Guangdong, China

**Keywords:** miR-1611, lncRNA-Six1, *Six1*, myoblast proliferation, myoblast differentiation, transformation of muscle fiber types

## Abstract

Emerging studies indicate important roles for non-coding RNAs (ncRNAs) as essential regulators in myogenesis, but relatively less is known about their function. In our previous study, we found that lncRNA-Six1 can regulate *Six1* in *cis* to participate in myogenesis. Here, we studied a microRNA (miRNA) that is specifically expressed in chickens (miR-1611). Interestingly, miR-1611 was found to contain potential binding sites for both lncRNA-Six1 and *Six1*, and it can interact with lncRNA-Six1 to regulate *Six1* expression. Overexpression of miR-1611 represses the proliferation and differentiation of myoblasts. Moreover, miR-1611 is highly expressed in slow-twitch fibers, and it drives the transformation of fast-twitch muscle fibers to slow-twitch muscle fibers. Together, these data demonstrate that miR-1611 can mediate the regulation of Six1 by lncRNA-Six1, thereby affecting proliferation and differentiation of myoblasts and transformation of muscle fiber types.

## 1. Introduction

Since the implementation of the Human Genome Project, scientists have been trying to explore the biological significances of genomic nucleic acid sequences. Protein coding genes only account for a small portion (1.5%) of the genome, while more than 85% of the genome is transcribed to non-coding RNA (ncRNA) [1,2]. Myogenesis is a process including myoblast proliferation, differentiation and myotube formation and is controlled by a series of myogenic regulatory factors. These factors can regulate myoblasts to withdraw from the cell cycle, express muscle-specific genes, and prevent the expression of other cell- or tissue-specific genes. The muscle growth of chickens is a complex quantitative characteristic which is directly regulated by major genes. However, recent studies have found that ncRNA also plays an important role [3,4,5,6].

MicroRNAs (miRNAs) are endogenous noncoding single-stranded RNAs in the eukaryotic genome that are approximately 18–22 nt in length [7]. In animal genomes, miRNAs participate in approximately 30% of gene expression regulation [8,9], playing a critical role in the regulation of gene expression at posttranscriptional levels. Recent studies have found that miRNAs are widely involved in the regulation of muscle developmental processes in chickens [10,11,12]. MiR-1611 is a unique miRNA in chickens, which was first found in developing chicken embryos in 2008 [13]. However, the molecular function of miR-1611 has not been reported yet.

Long non-coding RNAs (lncRNAs) are a new class of regulatory RNAs with a length greater than 200 bp, which account for about 87% of the total number of noncoding RNAs [14,15]. Recent studies have demonstrated that lncRNAs play crucial roles in wide range of biological processes, and they can regulate gene expression via epigenetic alterations, as well as transcriptional and posttranscriptional processing [16,17,18,19]. However, lncRNA in chicken research is new, and the roles of lncRNAs in chickens are still poorly understood [20,21,22].

In 2011, competitive endogenous RNAs (ceRNAs) were first reported as endogenous sponges that can affect the distribution of miRNAs on their targets, thereby imposing another novel layer of posttranscriptional regulation [23]. Recent studies have found that lncRNAs can act as endogenous competitive RNAs that interact with miRNAs and participate in the regulation of target gene expression [24,25,26,27]. 

In our previous study, we found lncRNA (lncRNA-Six1), which is located upstream of the protein-coding *Six1* gene, could encode a micropeptide to activate *Six1* in *cis* [28]. To further understand regulation by lncRNA-Six1, we predicted that miRNA that can bind with it. Interestingly, by using in silico analysis, we found that miR-1611 contains binding sites for lncRNA-Six1 and *Six1*. Mechanistic investigations showed that lncRNA-Six1 could also function as a ceRNA by sponging miR-1611, thus activating *Six1*. miR-1611 functions to regulate myoblast proliferation and differentiation, and is involved in the formation of skeletal muscle fibers, which supports the idea of its regulation of lncRNA-Sx1 and *Six1*. This result further complements the understanding of molecular regulation of lncRNA in chicken skeletal muscle development, and provides theoretical support for lncRNA being responsible for regulating protein-encoding genes through multiple pathways. 

## 2. Materials and Methods

### 2.1. Ethics Statement

All animal experimental protocols in this study were sanctioned by the Institutional Animal Care and Use Committee at the South China Agricultural University (approval ID: SCAU#0011). All the experiments were performed according to the regulations and guidelines established by the Committee and International Standards for Animal Welfare.

### 2.2. Animals and Tissues

Seven-week-old Xinghua (XH) female chickens were hatched from the Avian Farm of South China Agricultural University (Guangzhou, China). The chickens were euthanized, and organs and tissues were collected after rapid dissection, then immediately frozen in liquid nitrogen and stored at −80 °C. 

### 2.3. Hematoxylin and Eosin (H&E) Staining

The muscle tissues were immersed in 4% paraformaldehyde, and then embedded in paraffin and cut into 4 μm thick transverse sections. Subsequently, the sections were stained with hematoxylin and eosin.

### 2.4. Cell Culture

Chicken primary myoblasts (CPMs) were isolated from E11 chicken leg muscles as previously described [12], and cultured in Roswell Park Memorial Institute (RPMI)-1640 medium (Gibco, Gaithersburg, MD, USA) with 20% fetal bovine serum (FBS; Gibco).

DF-1 cell line chicken embryo fibroblasts were cultivated in cell culture vessels containing Dulbecco’s modified Eagle’s medium (DMEM; Gibco) supplemented with 10% FBS (Gibco).

All cells were incubated at 37 °C with an atmosphere of 5% CO_2_.

### 2.5. RNA Extraction, cDNA Synthesis and Quantitative Real-Time PCR (qRT-PCR)

Total RNA was extracted from tissues or cells using TRIzol reagent (Invitrogen Life Technologies, Carlsbad, CA, USA) as recommended by the supplier. The integrity and quantity of RNA were assessed using 1.5% agarose gel electrophoresis and a spectrophotometer (ND-2000, NanoDrop Inc., Wilmington, DE, USA).

Complementary RNA (cDNA) synthesis for messenger RNA (mRNA) was carried out using the PrimeScript RT Reagent Kit with gDNA Eraser (Perfect Real Time) (TaKaRa, Otsu, Japan). For miRNA, BμLge-Loop™ miRNA qRT-PCR Primer specific for miR-1611 and U6 were designed by RiboBio (RiboBio, Guangzhou, China), and a ReverTra Ace qPCR RT Kit (Toyobo, Osaka, Japan) was used to synthesize cDNA.

A real-time qPCR with iTaq Universal SYBR Green Supermix Kit (Bio-Rad Laboratories Inc., Hercules, CA, USA) was used, and analysis was performed with the 2^−ΔΔCt^ method, as described previously [29]. Chicken *β-actin* and *U6* were used as internal controls. Primers for RT-qPCR are shown in Table S1.

### 2.6. RNA Oligonucleotide and Plasmid Construction

Gga-miR-1611 mimics and mimic negative control (NC), gga-miR-1611 inhibitor, inhibitor NC, and small interfering RNAs (siRNAs) used for the knockdown of *Six1* were designed and synthesized by RiboBio (Guangzhou, China). The siRNA and antisense oligonucleotide (ASO) that were used for the specific knockdown of lncRNA-Six1 in the cytoplasm and nucleus, respectively, and non-specific siRNA NC duplex, were also designed and synthesized. Oligonucleotide sequences in this study are shown in Table S2.

lncRNA-Six1 and *Six1* overexpression plasmids were constructed as in our previous study [28]. For the pmirGLO dual-luciferase miRNA target reporter vector, the segment sequence of lncRNA-Six1 and *Six1* 3′ untranslated region (UTR) that contained the putative miR-1611 binding sequence was amplified by PCR, and then subcloned into *Xho*I and *Sal*I restriction sites in the pmirGLO dual luciferase reporter vector (Promega, Madison, WI, USA). Mutant plasmids were generated by changing the binding site of miR-1611 from AGCCCTC to GATTTCT. The major primers used in this study are listed in Table S3.

### 2.7. Cell Transfection

All transient transfections were performed with Lipofectamine 3000 reagent (Invitrogen, USA) according to manufacturer’s directions. In this study, si-lncRNA-Six1 and ASO- lncRNA-Six1 were co-transfected for interference with lncRNA-Six1, and named as si-ASO- lncRNA-Six1.

### 2.8. Dual-Luciferase Reporter Assay 

Cells were seeded in a 96-well plate. After being co-transfected, (a) wild-type or mutant-type lncRNA-Six1 position 1 dual-luciferase reporter, (b) wild-type or mutant-type lncRNA-Six1 position 2 dual-luciferase reporter, and (c) wild-type or mutant-type *Six1*-3′ UTR dual-luciferase reporter, with miR-1611 mimic or mimic NC, the firefly and Renilla luciferase activities were measured at 48 h post-transfection using a Dual-GLO Luciferase Assay System Kit (Promega, USA), following the manufacturer’s instructions. The luminescent signal was quantified using a Fluorescence/Multi-Detection Microplate Reader (BioTek, Winooski, VT, USA), and firefly luciferase activities were normalized to Renilla luminescence in each well.

### 2.9. Western Blot

A Western blot analysis was performed using a method that has been described in detail previously [12]. The antibodies and their dilutions used in this assay were as follows: rabbit anti-Six1 (sc-9127; Santa Cruz, USA; 1:200), myogenin antibody (orb6492; Biorbyt, UK; 1:100), B103 (DHSB, USA; 0.5 μg/mL), rabbit anti-glyceraldehyde-3-phosphate dehydrogenase (GAPDH) (AB-P-R 001; Hangzhou Goodhere Biotechnology Co. Ltd., Hangzhou, China; 1:1500), MYH1A antibody (F59; DHSB, USA; 0.5 μg/mL), MYH7B antibody (S58; DHSB, USA; 0.5 μg/mL), goat anti-rabbit IgG–horseradish peroxidase (HRP) (BA1054; Boster, Wuhan, China; 1:5000) and peroxidase–goat anti-mouse IgG (BA1051; Boster, China; 1:2500).

### 2.10. Flow Cytometric Analysis of the Cell Cycle

After transfection for 48 h, the cultured cells in growth media were collected and fixed overnight in 70% ethanol at −20 °C. Subsequently, the fixed cells were stained with propidium iodide (Sigma, Louis, MO, USA) (50 μg/mL), and then incubated at 37 °C in the dark for 30 min. Flow cytometry analysis was performed on a BD AccuriC6 flow cytometer (BD Biosciences, San Jose, CA, USA), and the data was processed using FlowJo software (7.6, Treestar Incorporated, Ashland, OR, USA).

### 2.11. 5-Ethynyl-2’-Deoxyuridine (EdU) Assay

The primary myoblasts were exposed to 50 μM EdU (RiboBio, China) for 2 h at 37 °C after 48 h transfection. Subsequently, the cells were fixed in 4% paraformaldehyde for 30 min and neutralized using 2 mg/mL glycine solution, and then permeabilized by adding 0.5% Triton X-100. A solution containing EdU (Apollo Reaction Cocktail; RiboBio, China) was added, and the cells were incubated at room temperature for 30 min. The nuclear stain Hoechst 33,342 was then added, and incubation was continued for another 30 min. A fluorescence microscope (DMi8; Leica, German) was used to capture three randomly selected fields to visualize the number of EdU-stained cells.

### 2.12. CCK-8 Assay

Primary myoblasts were seeded in a 96-well plate and cultured in growth medium. After being transfected, cell proliferation was monitored using a TransDetect CCK (TransGen Biotech, Beijing, China), according to the manufacturer’s protocol. Absorbance was measured using a Model 680 Microplate Reader (Bio-Rad, Hercules, CA, USA) by optical density at a wavelength of 450 nm.

### 2.13. Immunofluorescence

The immunocytofluorescence was performed using anti-MyHC (B103; DHSB, USA; 2.5 μg/mL) and Goat Anti-Mouse IgG (H + L)—Dylight 594 (BS10027; Bioworld, USA; 1:100). Images were captured using a fluorescence microscope (DMi8; Leica, German). The area of cells labeled with anti-MyHC was measured and calculated as previously described [12].

For immunohistofluorescence, fixed muscle tissues were embedded in paraffin, and then cut into seiscrop sections. Rabbit anti-Six1 (sc-9127; Santa Cruz, CA, USA; 1:50), MYH1A antibody (F59; DHSB, USA; 5 μg/mL), MYH7B antibody (S58; DHSB, USA; 5 μg/mL), goat anti-rabbit IgG (H + L)—Dylight 594 (BS10029; Bioworld, USA; 1:100), goat anti-mouse IgG (H + L), AF488 conjugate (TransGen Biotech, China; 1:100), and goat anti-mouse IgG (H + L) PE conjugate (TransGen Biotech, China; 1:100) were used for labelling the signals. Images of signals in the slides were captured by a Leica DMi8 fluorescent microscope.

### 2.14. Statistical Analysis

All results are presented as the mean ± S.E.M, based on at least three independent experiments for each treatment. Statistical significance was determined by one-sample or paired *t*-tests.

## 3. Results

### 3.1. miR-1611 Regulates the Proliferation and Differentiation of Myoblasts, and Is Involved in the Formation of Skeletal Muscle Fibers

**Figure 1:**
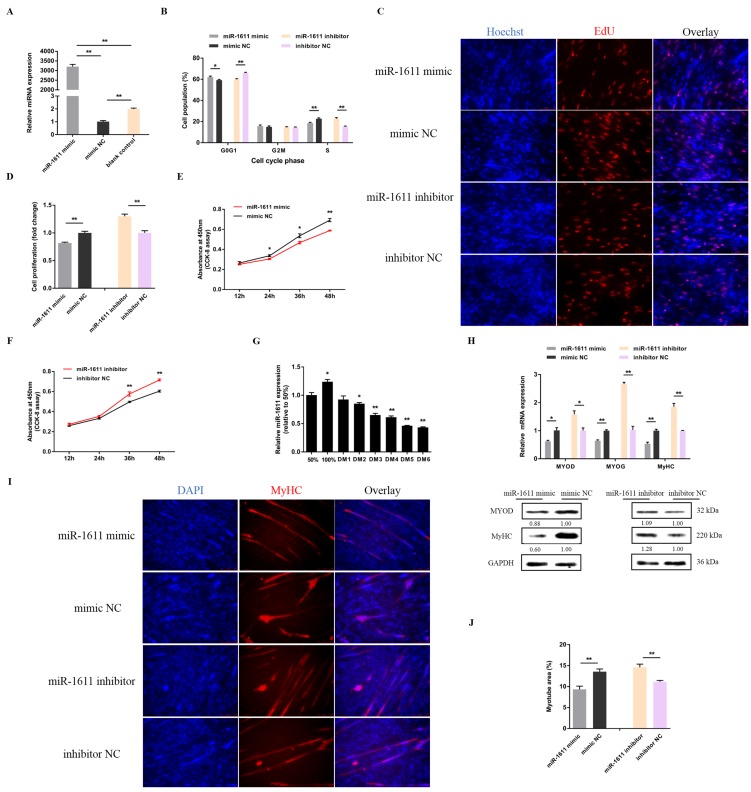
miR-1611 suppresses myoblast proliferation and differentiation. (**A**) The relative expression of miR-1611 after transfection with the indicated microRNA (miRNA) mimic. (**B**) Cell cycle analysis of myoblasts at 48 h after overexpression or inhibition of miR-1611. (**C**) Proliferation of transfected chicken primary myoblasts (CPMs) was assessed by 5-ethynyl-2’-deoxyuridine (EdU) incorporation. (**D**) Proliferation rate of myoblasts transfected with an miR-1611 mimic and inhibitor. (**E**—**F**) CCK-8 assays were performed in CPMs with miR-1611 overexpression and inhibition. (**G**) Relative miR-1611 expression during CPM differentiation. DM indicates the differentiation day. (**H**) The messenger RNA (mRNA) and protein expression levels of myoblast differentiation marker genes from miR-1611 mimic or inhibitor transfected CPMs. The numbers shown below the bands were folds of band intensities relative to control. Band intensities were quantified by ImageJ and normalized to glyceraldehyde-3-phosphate dehydrogenase (GAPDH). Data are expressed as a fold-change relative to the control. There was only one Western blot performed per treatment, and therefore n = 1. (**I**) MyHC immunostaining of CPMs transduced with a miR-1611 mimic or inhibitor. Cells were differentiated for 72 h after transfection. The nuclei were visualized with 4′,6-diamidino-2-phenylindole (DAPI). (**J**) Myotube area (%) of CPMs 72 h after the overexpression and inhibition of miR-1611. In all panels, data are presented as mean ± SEM of three biological replicates. Statistical significance of differences between means was assessed using an unpaired Student’s *t*-test. (* *p* < 0.05; ** *p* < 0.01) vs. NC, negative control.

In order to unveil the functions of miR-1611, we performed overexpression and inhibition experiments to assess its role in myoblast proliferation and differentiation. After 48 h of transfection with a miR-1611 mimic, the relative expressions of miR-1611 were detected (Figure 1A). Overexpression of miR-1611 led to a significant increase of the number of cells that progressed to G0/G1, and that reduced the number of S phase cells (Figure 1B). Conversely, miR-1611 inhibition resulted in a smaller number of G0/G1, and increased the number S phase cells (Figure 1B). Furthermore, using EdU and CCK-8 assays, we found that miR-1611 overexpression significantly repressed myoblast proliferation (Figure 1C–E). On the contrary, its loss of function induces cell division (Figure 1C,D,F). To further investigate the potential roles of miR-1611 in differentiation, we detected the relative expressions of miR-1611 after inducing the CPMs to differentiate in vitro (Figure 1G). The expression level of miR-1611 showed a gradual decrease; hence, it is suggested that miR-1611 may play an important role in the process of myoblast differentiation. In the meantime, we also detected the expressions of myoblast differentiation marker genes, such as *MYOD*, *MYOG*, and *MyHC*, after overexpression or inhibition of miR-1611 (Figure 1H). miR-1611 overexpression suppressed muscle differentiation marker gene expression, whereas the expressions of these genes were significantly induced after miR-1611 inhibition. Moreover, immunofluorescence staining also showed that the overexpression of miR-1611 could promote myoblast differentiation and induce myotube formation (Figure 1I,J). On the contrary, the total area of myotube was significantly increased with the inhibition of miR-1611 (Figure 1I,J).

In poultry, breast muscle is generally considered to be composed of fast-twitch muscle fibers with a larger muscle fiber diameter, while the leg muscles are mainly composed of slow-twitch muscle fibers with a smaller muscle fiber diameter [30,31,32]. Using H&E staining, we found that breast muscle has a larger area and diameter than leg muscle (Figure S1A–C). Moreover, we also evaluated some of specific myosin heavy chain isoforms (for example, MYH1A for fast-twitch muscle fibers and MYH7B for slow-twitch muscle fibers) by immunohistofluoresence staining and Western blot (Figure S1D–F). The result showed that MYH1A is highly expressed in breast muscle, while the expression of MYH7B is higher in leg muscle.

**Figure 2:**
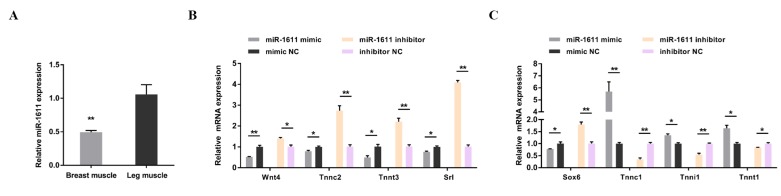
miR-1611 participates in the formation of skeletal muscle fibers. (**A**) Relative expression of miR-1611 in the breast muscles and leg muscles of 7-week-old Xinghua (XH) chicken. (**B**,**C**) mRNA levels of several fast muscle genes and slow muscle genes induced by miR-1611 overexpression and inhibition in CPMs. In all panels, the results are shown as mean ± SEM, and the data are representative of three independent assays. Statistical significances of differences between means were assessed using an unpaired Student’s *t*-test. (* *p* < 0.05; ** *p* < 0.01) vs. NC, negative control.

Tissue expression profiles for miR-1611 showed a differential expression between breast muscle and leg muscle, leading to speculation that miR-1611 may function in the formation of skeletal muscle fiber (Figure 2A). To further investigate the role of miR-1611 during the transformation of skeletal muscle fiber types, we detected the expression of a series of fast muscle genes (including *Wnt4*, *Tnnc2*, *Tnnt3*, and *Srl*) and a series of slow muscle genes (including *Sox6*, *Tnnc1*, *Tnni1*, and *Tnnt1*) (Figure 2B,C). Overexpression of miR-1611 downregulated the expression of fast muscle genes and promoted slow muscle gene expression. Inhibition of its function drove the transformation of slow-twitch muscle fibers to fast-twitch muscle fibers.

### 3.2. miR-1611 Is a Direct Target of the ceRNA Network between lncRNA-Six1 and Six1

**Figure 3:**
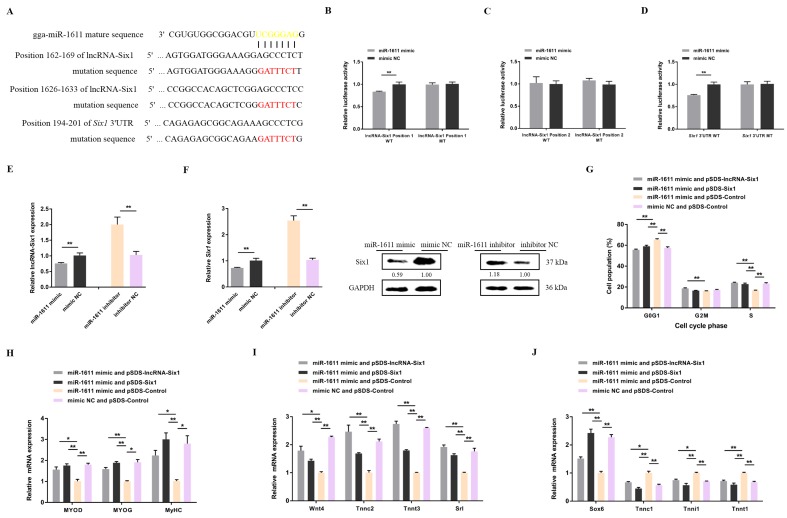
miR-1611 interacts with lncRNA-Six1 to regulate *Six1* expression. (**A**) The potential binding site of miR-1611 in the lncRNA-Six1 transcript and the *Six1* 3′ untranslated region (UTR). The mutant sequence in the miR-1611 binding site is highlighted in red. (**B**–**D**) A dual-luciferase reporter assay was conducted by co-transfecting the wild type or mutant: (**B**) lncRNA-Six1 position 1, (**C**) lncRNA-Six1 position 2, or (**D**) *Six1* 3′ UTR with a miR-1611 mimic or mimic-NC in DF-1 cells. (**E**) Relative lncRNA-Six1 expression after overexpression and inhibition of miR-1611. (**F**) The mRNA and protein expression levels of *Six1* from the miR-1611 mimic and inhibitor-transfected CPMs. The numbers shown below the bands were folds of band intensities relative to the control. Band intensities were quantified by ImageJ and normalized to GAPDH. Data are expressed as a fold-change relative to the control. There is only one Western blot performed per treatment, and therefore, n = 1. (**G**) Cell cycle analysis of CPMs after co-transfection with the listed nucleic acids. (**H**) Relative mRNA levels of several myoblast differentiation marker genes from co-transfected CPMs. (**I**,**J**) The mRNA expression levels of several fast and slow muscle genes induced by the listed nucleic acids in CPMs. In all panels, data are presented as means ± S.E.M. of three independent experiments, and the statistical significance of differences between means was assessed using an unpaired Student’s *t*-test. (* *p* < 0.05; ** *p* < 0.01) vs. NC, negative control.

To further investigate the molecular mechanism of how miR-1611 exerts its biological effect, we predicted its target lncRNAs and genes on an RNAhybrid (https://bibiserv.cebitec.uni-bielefeld.de/rnahybrid) [33]. Interestingly, miR-1611 was found to contain potential binding sites for both lncRNA-Six1 and *Six1*, implying a new mechanism by which lncRNA-Six1 regulates *Six1* (Figure 3A). Dual-luciferase reporter assays were carried out to confirm whether miR-1611 can directly interact with lncRNA-Six1 and *Six1* (Figure 3B–D). The results showed that miR-1611 could perfectly bind with and target to interact with both lncRNA-Six1 position 1 and the 3′ UTR of *Six1*. More importantly, overexpression of miR-1611 significantly decreased the expression level of lncRNA-Six1 and *Six1*, whereas the expression of lncRNA-Six1 and *Six1* were upregulated with the inhibition of miR-1611 (Figure 3E,F). In addition, the effects of miR-1611 on myoblast proliferation and differentiation, and the formation of skeletal muscle fibers, were negated with the co-overexpression with lncRNA-Six1 or *Six1* (Figure 3G–J). Taken together, these results demonstrated that lncRNA-Six1 can function as a ceRNA for miR-1611 to regulate *Six1*.

### 3.3. lncRNA-Six1 Functions to Promote Myoblast Proliferation and Differentiation, and Induces the Fast-Twitch Muscle Phenotype

**Figure 4:**
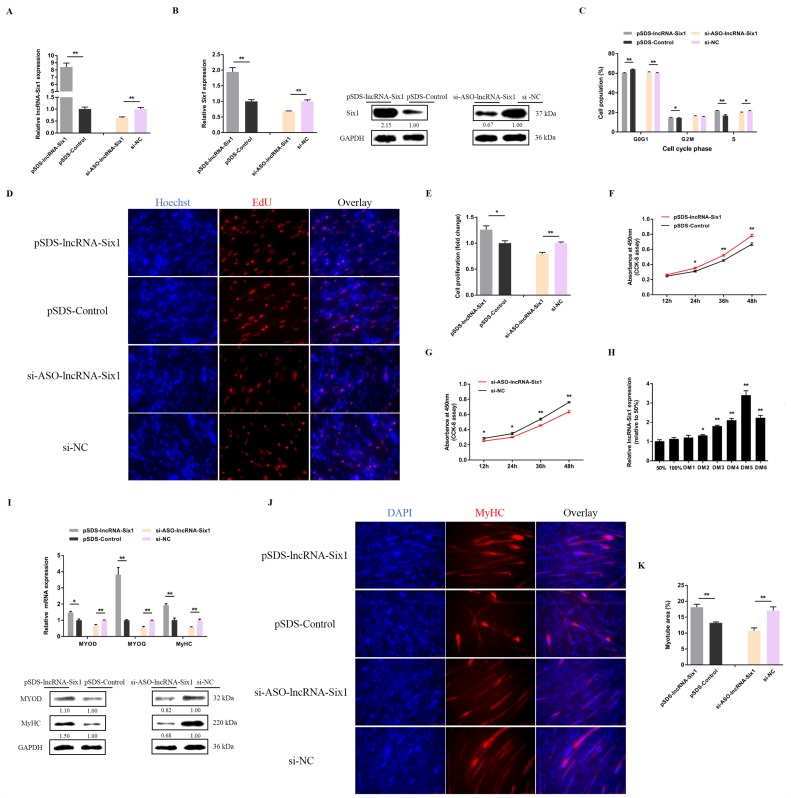
lncRNA-Six1 functions to facilitate myoblast proliferation and differentiation. (**A**) The relative expression levels of lncRNA-Six1 with lncRNA-Six1 overexpression and knockdown. (**B**) The mRNA and protein expression levels of *Six1* from pSDS-lncRNA-Six1 and si-ASO-lncRNA-Six1-transfected CPMs. The numbers shown below the bands were folds of band intensities relative to the control. Band intensities were quantified by ImageJ and normalized to GAPDH. Data are expressed as a fold change relative to the control. There was only one Western blot performed per treatment, and therefore n = 1. (**C**) Cell cycle analysis of CPMs after lncRNA-Six1 overexpression and knockdown. (**D**) EdU proliferation assays for CPMs with the overexpression and inhibition of lncRNA-Six1, (**E**) the numbers of proliferative cells were also counted. (**F**,**G**) CPM growth curves following the transfection of pSDS-lncRNA-Six1 and si-ASO-lncRNA-Six1. (**H**) Relative lncRNA-Six1 expression during CPM differentiation. (**I**) The mRNA and protein expression levels of myoblast differentiation marker genes with lncRNA-Six1 overexpression and knockdown in the CPMs. The numbers shown below the bands were fold-changes of band intensities relative to control. Band intensities were quantified by ImageJ and normalized to GAPDH. Data are expressed as a fold-change relative to the control. There was only one Western blot performed per treatment, and therefore n = 1. (**J**) Immunofluorescence analysis of MyHC-staining cells with the overexpression and knockdown of lncRNA-Six1 in CPMs. (**K**) Myotube area (%) of CPMs with lncRNA-Six1 overexpression and knockdown. In all panels, results are expressed as the mean ± SEM of three independent experiments, and the statistical significance of differences between means was assessed using an unpaired Student’s *t*-test. (* *p* < 0.05; ** *p* < 0.01) vs. NC, negative control.

lncRNA-Six1 overexpression and knockdown experiments were performed to verify the biological functions of lncRNA-Six1 in myoblasts (Figure 4A). Overexpression of lncRNA-Six1 upregulated the mRNA and protein expression level of *Six1*, while knockdown of lncRNA-Six1 inhibited *Six1* expression (Figure 4B). Meanwhile, lncRNA-Six1 overexpression resulted in a decrease in G0/G1 phase cells, and an augmented number of cells that progressed to the S phase (Figure 4C), as well as significantly promoting myoblast proliferation (Figure 4D–F). However, G0/G1 cells increased, while S phase cells decreased significantly (Figure 4C), and myoblast proliferation was significantly suppressed with lncRNA-Six1 interference (Figure 4D,E,G). In addition, the expression of lncRNA-Six1 was upregulated after the induction of CPM differentiation in vitro (Figure 4H). The transfection of pSDS-lncRNA-Six1 induced the expression of the myoblast differentiation marker gene, while the interference of lncRNA-Six1 downregulated its expression (Figure 4I). Immunocytofluorescence staining showed that lncRNA-Six1 overexpression facilitated myoblast differentiation, which was associated with increased total areas of myotubes (Figure 4J). In contrast, the differentiation of myoblasts was significantly restrained with lncRNA-Six1 knockdown (Figure 4K).

**Figure 5:**
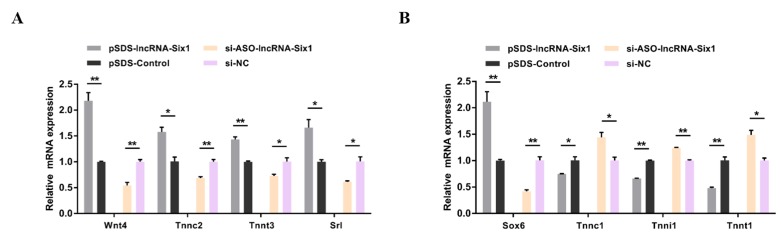
lncRNA-Six1 induces the fast-twitch muscle phenotype. (**A**,**B**) The relative mRNA expression of several fast and slow muscle genes induced by lncRNA-Six1 overexpression and inhibition in CPMs. In all panels, data are presented as means ± S.E.M. of three independent assays. Statistical significance of differences between the means was assessed using an unpaired Student’s *t*-test. (* *p* < 0.05; ** *p* < 0.01) vs. NC, negative control.

To evaluate the role of lncRNA-Six1 in the muscle fiber phenotype, we assayed the expressions of fast and slow muscle genes after lncRNA-Six1 overexpression or knockdown (Figure 5A,B). The expressions of multiple fast-type muscle genes were significantly promoted by lncRNA-Six1 overexpression, while the interference of lncRNA-Six1 activated the slow muscle program.

### 3.4. Six1 Is a Critical Factor in Myogenesis, Participating in the Transformation of Skeletal Muscle Fiber Types

**Figure 6:**
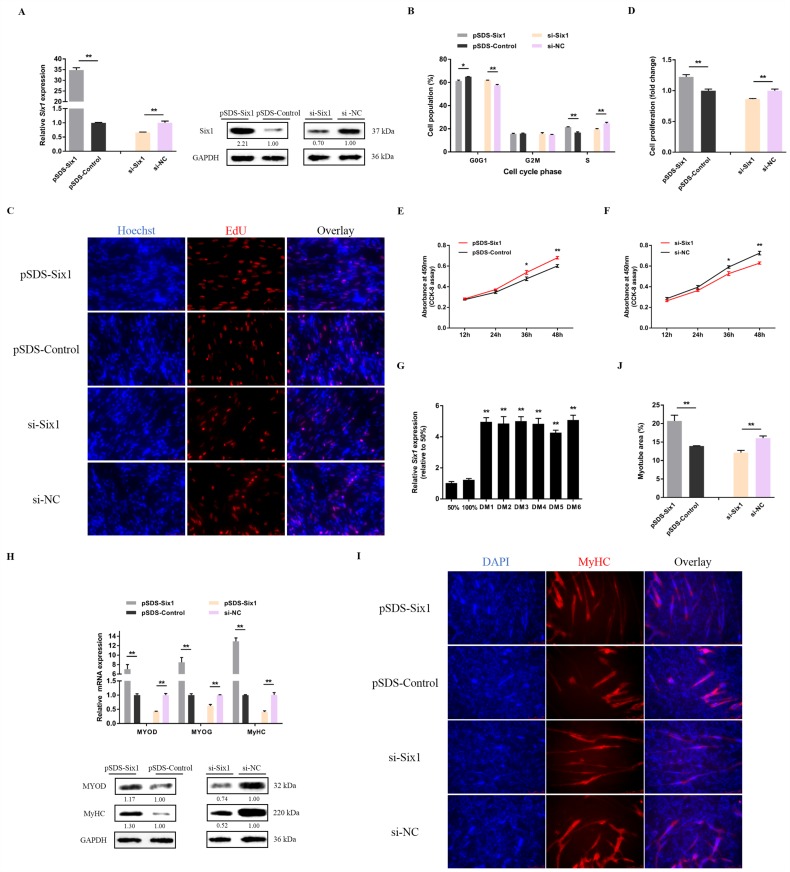
*Six1* is involved in the proliferation and differentiation of myoblasts. (**A**) The mRNA and protein expression levels of *Six1* with *Six1* overexpression and knockdown in CPMs. The numbers shown below the bands were folds of band intensities relative to the control. Band intensities were quantified by ImageJ and normalized to GAPDH. Data are expressed as a fold-change relative to the control. There was only one Western blot performed per treatment, and therefore n = 1. (**B**) CPMs were collected for cell cycle analysis 48 h after transfection. (**C**) EdU proliferation assays for CPMs with the overexpression and inhibition of *Six1*, (**D**) the numbers of proliferative cells were also counted. (**E**,**F**) Cell growth was measured after *Six1* overexpression and knockdown. (**G**) The relative mRNA expression level of *Six1* during CPM differentiation. (**H**) Relative mRNA and protein expression of the differentiation marker genes after transfection with pSDS-Six1 and si-Six1. The numbers shown below the bands are fold-changes of band intensities relative to the control. Band intensities were quantified by ImageJ and normalized to GAPDH. Data are expressed as a fold-change relative to the control. There was only one Western blot performed per treatment, and therefore n = 1. (**I**) MyHC staining of myoblasts at 72 h after overexpression and inhibition of *Six1*. (**J**) Myotube area (%) of CPMs with *Six1* overexpression and knockdown. In all panels, results are expressed as the mean ± SEM of three replicates. Statistical significance of differences between means was assessed using an unpaired Student’s *t*-test. (* *p* < 0.05; ** *p* < 0.01) vs. NC, negative control.

To determine the function of *Six1* in myogenesis, we constructed a *Six1* overexpression vector and synthesized the specific siRNA (Figure 6A). *Six1* overexpression led to a decrease in the number of G0/G1 cells, and increased the number of cells in the S phase (Figure 6B), as well as significantly facilitated myoblast proliferation as judged by EdU incorporation and CCK-8 assay (Figure 6C–E). Inversely, the opposite results were shown with *Six1* knockdown (Figure 6B–D,F). During myoblast differentiation, the expression of *Six1* was significantly upregulated (Figure 6G). At the same time, the expression of myoblast differentiation marker genes was found to be significantly promoted after the overexpression of *Six1*, whereas the interference of *Six1* restrained its expression (Figure 6H). Furthermore, *Six1* overexpression significantly promoted the formation of myotubes and increased the size of the myotube area (Figure 6I,J). In contrast, *Six1* interference suppressed myoblast differentiation (Figure 6I,J).

**Figure 7:**
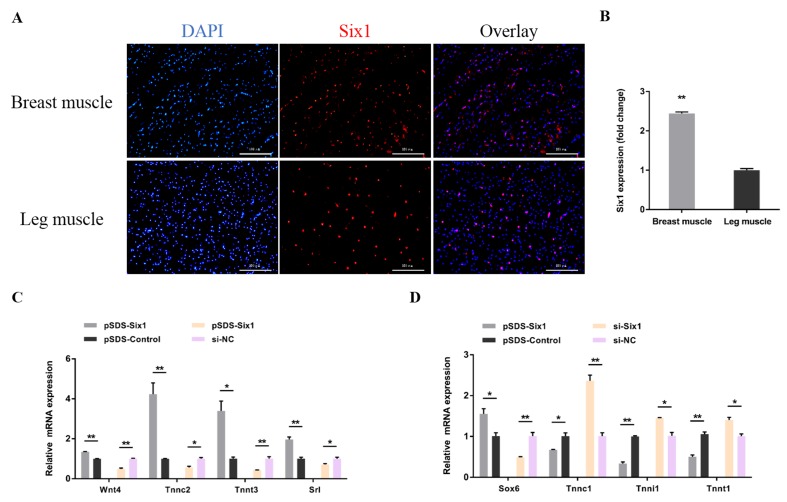
*Six1* drives the transformation of skeletal muscle fiber types. (**A**) Immunofluorescence analysis of Six1-staining cells of breast muscles and leg muscles in 7-week-old XH chicken. (**B**) Relative *Six1* expression in breast muscles and leg muscles of 7-week-old XH chicken. (**C**,**D**) The mRNA expression levels of several fast and slow muscle genes after *Six1* overexpression and knockdown in CPMs. In all panels, results are expressed as the mean ± S.E.M. of three biological replicates. Statistical significance of the differences between means was assessed using an unpaired Student’s *t*-test. (* *p* < 0.05; ** *p* < 0.01) vs. NC, negative control.

In a previous study, we identified the tissue expression profile of *Six1* by qRT-PCR [28]. Compared with leg muscles, we also found by immunohistofluorescence that *Six1* is expressed more in breast muscle (Figure 7A,B). Overexpression of *Six1* significantly upregulated the fast muscle genes, and downregulated the slow muscle genes, while the inverse results were observed with the knockdown of *Six1* (Figure 7C,D). Taken together, we argue that *Six1* plays a critical role, not only in myoblast development, but also in the transformation of muscle fibers from one type to the other in skeletal muscle development.

## 4. Discussion

**Figure 8:**
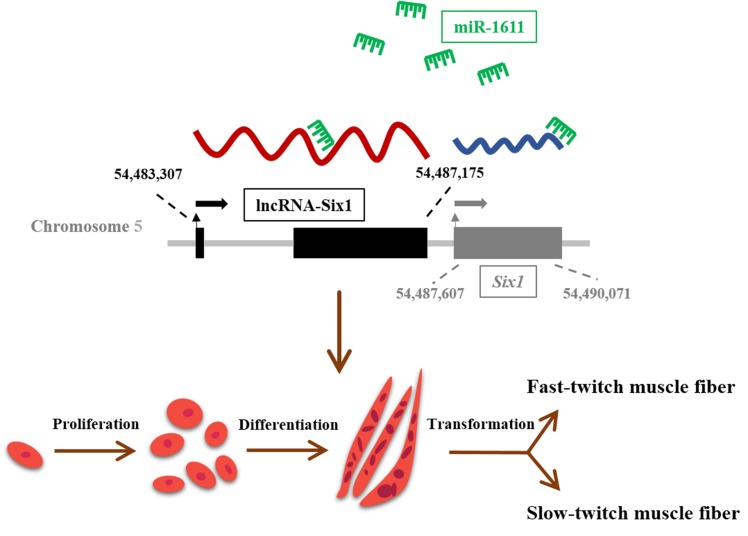
Graphical abstract of miR-1611 regulation in myogenesis.

The present study reveals a role of miR-1611 in the proliferation and differentiation of myoblasts, as well as the transformation of skeletal muscle fiber types. Our findings are partly based on the function of miR-1611 in directly interacting with lncRNA-Six1 and *Six1* (Figure 8).

miRNAs are well-known to function as important regulators and mediators in myogenesis. In chickens, many miRNAs were found to widely participate in muscle developmental processes in several different ways [5,10,11,12,34]. In this study, we first analyzed the function of miR-1611 in myogenesis, which is specifically expressed in chickens. Overexpression of miR-1611 repressed the proliferation and differentiation of myoblasts. In addition, miR-1611 is highly expressed in slow-twitch fibers, and it could active the slow muscle program.

Recently, a new pattern of gene expression has been put forward regarding the interaction of RNA transcripts, referred to as ceRNAs [23]. There is evidence that lncRNAs can function as ceRNAs to protect mRNAs by acting as molecular sponges for miRNAs, thereby modulating the de-repression of miRNA targets and imposing an additional level of posttranscriptional regulation [35,36,37]. In the current study, using in silico analysis, we identified that miR-1611 contains binding sites for lncRNA-Six1 and *Six1*. A dual-luciferase reporter assay validated the direct binding of the miR-1611 response elements on lncRNA-Six1 transcript and *Six1* 3′ UTR. Moreover, our rescue experiment showed that the inhibition effect of miR-1611 in regulation of *Six1* could be neutralized when lncRNA-Six1 is overexpressed. These data indicated that miR-1611 is a crucial mediator for lncRNA-Six1 to function as a ceRNA, thus regulating *Six1*.

It is worth noting that lncRNAs have also been demonstrated to function during muscle growth [38,39]. For instance, the noncoding RNA steroid receptor RNA activator (SRA) was reported to be an enhancer of the myogenic differentiation and myogenic conversion of non-muscle cells, through the co-activation of MyoD activity [40,41]. Moreover, lnc-MD1 [24], Yam-1 [42], H19 [25,35,43], and Malat1 [26] are also believed to be important regulators of myogenesis. In our study, we identified that lncRNA-Six1 could regulate the expression of *Six1* by acting as a ceRNA. lncRNA-Six1 overexpression significantly promoted the proliferation of myoblasts, and this induced myoblast differentiation. At the same time, lncRNA-Six1 is involved in the formation of skeletal muscle fibers, and it can promote the conversion of the muscle fiber phenotype.

*Six1* is an evolutionarily, highly conserved transcription factor that is widely involved in skeletal muscle development [44,45,46]. Recent research has shown that *Six1* determined proliferation and differentiation of the muscle lineage by targeting the myogenic regulatory factor family [44]. Moreover, *Six1* was also reported to be able to drive the transformation of slow-twitch fibers to the fast-twitch phenotype in skeletal muscle [44,47]. In the current study, we found *Six1* facilitated myoblast proliferation and differentiation. Furthermore, overexpression of *Six1* could upregulate fast muscle genes, downregulate slow muscle genes, and induce the transformation of slow-twitch muscle fibers to fast-twitch muscle fibers.

Altogether, the results of our study demonstrate that miR-1611 can act as a regulator to mediate the regulation of *Six1* by lncRNA-Six1, thus affecting myoblast proliferation and differentiation, as well as being involved in the transformation of skeletal muscle fiber types.

## Supplementary Materials

The Supplementary Materials are available online.
