# Supplementary material for: lncRNA-Six1 Is a Target of miR-1611 That Functions as a ceRNA to Regulate Six1 Protein Expression and Fiber Type Switching in Chicken Myogenesis
Source: Cells. 2018 Dec 4;7(12):243. doi: 10.3390/cells7120243 (PMC6315877; doi:10.3390/cells7120243)

Table S1. Primers used for real-time PCR.

Gene name	Primer sequences (5′ to 3′)	Annealing temperature (◦C)	Accession Number
lncRNA-Six1	F: CTCGTGAGAAGGGGGCAAAA<br>R: GAAGGCGAAACCCAAAACCG	56	-
Six1	F: GTCAGCAACTGGTTCAAGA<br>R: AGGAGGACCGAGTTCTGAT	59	NM_001044685.1
MYOD	F: GCTACTACACGGAATCACCAAAT<br>R: CTGGGCTCCACTGTCACTCA	53	NM_204214.2
MYOG	F: CGGAGGCTGAAGAAGGTGAA<br>R: CGGTCCTCTGCCTGGTCAT	53	NM_204184.1
MyHC	F: CTCCTCACGCTTTGGTAA<br>R: TGATAGTCGTATGGGTTGGT	53	NM_001319304.1
Wnt4	F: GGAGGCAGCGTTCGTCTA<br>R: GGCAATGTTATCGGAGCAG	56	NM_204783.1
Tnnc2	F: GAGCAGCAAAGATGGCGTCA<br>R: ATCACCGTGCCCAACTCCTT	57	NM_205450.2
Tnnt3	F: AGAGGGAAGAAGCAAACAGC<br>R: GTCCCACAGTTCCTTAGCCT	57	NM_204922.2
Srl	F: CCTCCTCGGGCTGGATGACA<br>R: GTTCTTGCTGCTTGCGGTTT	56	NM_205329.1
Sox6	F: TCAGGTTCAGGGTCACATGCC<br>R: TTGCTGGAGCTGTAAAGGGC	56	NM_001318451.1
Tnnc1	F: GTTGAGCAGTTGACAGAAGA<br>R: GAACCATCATAACAAGGAAC	57	NM_205133.1
Tnni1	F: GAGGAGTGGGAGCAGGAGAT<br>R: TTCGTCCACAATCTCAACCT	57	XM_004934839.2
Tnnt1	F: GAGCCGCACGGAGAAGGAGC<br>R: CCCGAAGTGGGGCATGTTGG	57	NM_205114.1
β-actin	F:GATATTGCTGCGCTCGTTG<br>R:TTCAGGGTCAGGATACCTCTTT	50-65	NM_205518.1

Table 2. Oligonucleotide sequences in this study.

Fragment name	Sequences (5′ to 3′)
miR-1611 mimic	GGAGGGCUUGCAGGCGGUGUGC
miR-1611 inhibitor	GCACACCGCCUGCAAGCCCUCC
si-lncRNA-Six1	GCTTATCTCAGCGCGTATA
ASO-lncRNA-Six1	TTAGCAGACACGCGGTCACG
si-*Six1*	GGGAGAACACGGAGAACAA

Table S3. Primers used for vector construction.

Primer name	Primer sequences (5′ to 3′)
pSDS-lncRNA-Six1	F: GGGG**GGTCTCTAGTG**GCCTGCTCCTGCAGCCCC<br>R: GCCG**GGTCTCGTGGG**TTTTTTTCCTTTTTTCCTTTTT
pSDS-Six1	F: GGGG**GGTCTCTAGTG**ATGTCGATGCTGCCGTCGTTCG<br>R: GCCG**GGTCTCGTGGG**CGTTCAGGATCACCGTCCGCTA
lncRNA-Six1 position 1 WT	F: CCG**CTCGAG**CCTCGGGTCGTGGCAAAT<br>R: ACGC**GTCGAC**CGGCCTTAGAAATAAGCCTACC
lncRNA-Six1 position 1 MT	F: CAGCAAGTGGATGGGAAAGGCTAAAGATCCAACACAAACCTC<br>R: GAGGTTTGTGTTGGATCTTTAGCCTTTCCCATCCACTTGCTG
lncRNA-Six1 position 2 WT	F: CCG**CTCGAG**CAACATCACAAGCACTAAAA<br>R: ACGC**GTCGAC**TGAGGGTGTCTTTATGGCA
lncRNA-Six1 position 2 MT	F: CACCGCCGGCCACAGCTCGGCTAAAGACCCGGCCGGGCCGTG<br>R: CACGGCCCGGCCGGGTCTTTAGCCGAGCTGTGGCCGGCGGTG
Six1 3′ UTR WT	F: CCG**CTCGAG**TGGACACGGACTCTCGGTT<br>R: ACGC**GTCGAC**ATTTATGGGGCGCAATGG
Six1 3′ UTR MT	F: AAATGCAGAGAGCGGCAGAACTAAAGAGGCCTTTCGAGTTCA<br>R: TGAACTCGAAAGGCCTCTTTAGTTCTGCCGCTCTCTGCATTT

Sequences in bold represent the enzyme cutting sites.

Figure S1. Identification of muscle fiber types between breast muscle and leg muscle. (**A**) H&E staining of transverse sections of breast muscles and leg muscles from 7-week-old XH chickens. (**B**) Muscle fiber area of breast muscles and leg muscles of 7-week-old XH chickens. (**C**) Muscle fiber diameter of breast muscles and leg muscles of 7-week-old XH chickens. (**D**) Immunofluorescence analysis of MYH1A/MYH7B-staining cells of breast muscles and leg muscles in 7-week-old XH chickens. (**E**) Relative MYH1A/MYH7B expression in breast muscles and leg muscles of 7-week-old XH chickens. (**F**) Relative protein expression of MYH1A and MYH7B in breast muscles and leg muscles of 7-week-old XH chickens. The numbers shown below the bands were fold-changes of band intensities relative to the control. Band intensities were quantified by ImageJ and normalized to GAPDH. Data are expressed as a fold change relative to the control. There was only one western blot performed per treatment, and therefore n = 1. In all panels, results are expressed as the mean ± S.E.M. of three replicates. Statistical significance of differences between means was assessed using an unpaired Student's *t*-test. (* $P < 0.05$; ** $P < 0.01$) vs. NC, negative control.

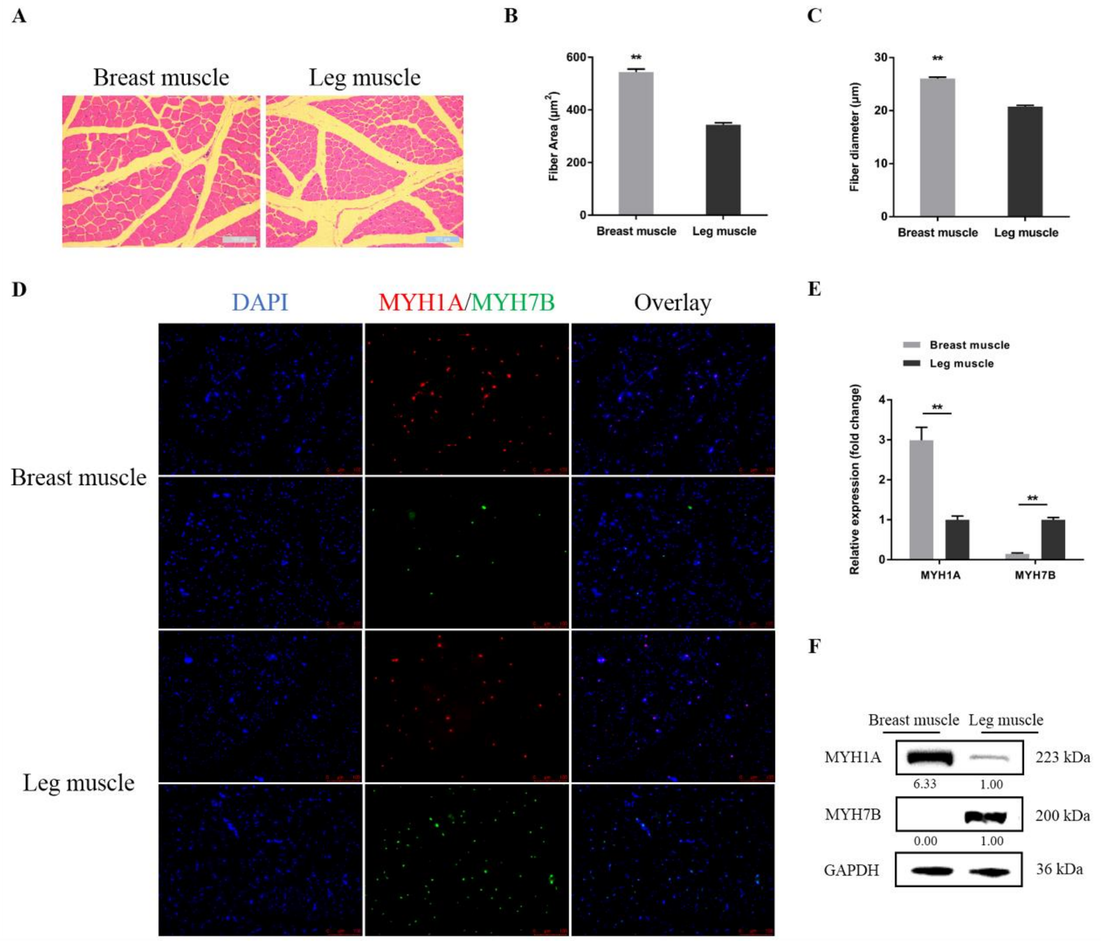

Supplement: Supplementary file 1 [file cells-07-00243-s001.pdf]
